# Species A Rotavirus (RVA) Isolated from Sewage in Nigeria, 2014: Close Genetic Relatedness of Partial G, P, and NSP4 Gene Sequences Encoding G1 with Cogent Genes of Other Asian and African Rotaviruses

**DOI:** 10.1155/2018/8425621

**Published:** 2018-06-24

**Authors:** Babatunde Olanrewaju Motayo, Johnson Adekunle Adeniji, Adedayo Omotayo Faneye

**Affiliations:** ^1^Department of Virology, College of Medicine, University of Ibadan, Ibadan, Nigeria; ^2^Medical Microbiology Unit, Pathology Department, Federal Medical Centre, Abeokuta, Nigeria

## Abstract

Rotavirus has been identified as a major cause of gastroenteritis in Nigeria. There is limited information on the intragenotype diversity of Nigerian rotavirus isolates. We therefore investigated the molecular characteristics of some rotavirus gene sequences detected in sewage from Nigeria. Seven sewage samples, out of a total of 68, tested positive for rotavirus RNA (10.3%). Genotype G1P[4] was the most common genotype (5 isolates) and one isolate for genotypes G1P[8] and G3P[6]. Phylogenetic analysis of the partial VP7 gene of 3 G1P[4] isolates analyzed identified them as genotype G1 Lineage 2 along with Chinese strains with 99.1% to 100% amino acid similarity. Amino acid substitutions D-97→E and S-147→D/N were observed within the 7-1a and 7-2 domains of VP7 gene among the study G1P4 isolates in reference to vaccine strain RotaTeq®. Phylogenetic analysis of the G3P[6] study isolate identified it as genotype G3 Lineage 3, forming a monophyletic cluster with 100% bootstrap value with other West African strains G3 isolates. Phylogenetic analysis of GIP[4] VP4 genes identified them as P4 Lineage 5, while 3 NSP4 gene sequences belonged to genotype E1, while 1 belonged to E2. The results from this study represent phylogenetic analysis of partial gene sequences of environmental group A rotavirus (RVA) isolates from Nigeria.

## 1. Introduction

Rotavirus is the most important agent of viral gastroenteritis in children [1, 2]. The virus is responsible for about 200,000 deaths in children below 5 years of age annually in low income countries [1, 3]. Rotaviruses are members of the virus family Reoviridae, possessing a double stranded (ds) RNA genome segmented into 11 compartments, coding for six structural proteins (VP1 to VP4, VP6, and VP7) and five or six nonstructural proteins (NSP1 to NSP5/6) [4]. Based on this structural organization at least 10 distinct species/groups (A-I, J) are differentiated based on their VP6 coding sequences [5, 6]. Group A rotavirus is responsible for the majority of human infections [4, 7, 8]. The major proteins capable of eliciting neutralizing antibodies against rotavirus challenge are the 34kDa glycoprotein VP7 and the 88kDa nonglycosylated spike protein VP4. These 2 proteins have been used to form a binary classification scheme for rotaviruses in analogy to that used for influenza virus classification [7].

Molecular epidemiology has identified 32 G (VP7) genotypes and 47 P (VP4) genotypes https://rega.kuleuven.be/cev/viralmetagenomics/virus-classification/7th-RCWG-meeting, update of the Rega Institute, KU Leuven, Belgium. Owing to the rapid evolution and high genetic diversity of rotavirus, a revised classification system was introduced which differentiated the complete genome sequence of rotavirus [9, 10]. In Nigeria, several studies have reported various genotypes including some unusual genotypes [11–15]. Recently, the presence of G12 strains was reported in Nigeria [15]. We also recently reported rotavirus G9 genotypes from sewage in Nigeria [16]. In Nigeria, the predominant rotavirus genotype has remained the G1P[4] and G1P[8] [14, 16].

Rotavirus A (RVA) contaminated sewage water has previously caused outbreaks of viral gastroenteritis [17]. Several reports have also shown the importance of sewage as a major contributor to rotavirus environmental dissemination [17–19]. Molecular characterization of rotavirus from sewage has also been shown to serve as an economical way of conducting molecular surveillance of rotavirus [20]. Our current study investigates the molecular characteristics and intragenotype diversity of partial VP7, VP4, and NSP4 genes of RVAs isolated from sewage water in Nigeria in 2014.

## 2. Materials and Methods

### 2.1. Sample Collection and Processing

Sixty-eight sewage effluent samples were collected from selected sites in Northern Nigeria between August and October 2014, by grab method in a white 1-liter plastic keg and transported in reverse cold chain in a geostyle box with ice packs. Sewage was concentrated using polyethylene glycol PEG 6000 and dextran 20% two-phase concentration method following World Health Organization (WHO) protocol [21]. Briefly centrifuge raw sewage sample was for 10 min at 1000 g. Pool supernatants in a 1-liter Erlenmeyer flask. To 500 ml of the supernatant, add 39.5 ml of 22% dextran, 287 ml 29% PEG 6000, and 35 ml 5N NaCl. Mix thoroughly and keep in constant agitation for 1 hour at 4°C using a horizontal shaker or magnetic stirrer. Pour the mixture into a separation funnel and leave overnight at 4°C, and carefully collect lower layer and the interphase drop-wise, into a sterile tube.

### 2.2. RT-PCR and Seminested PCR Genotyping

Viral RNA was extracted from concentrated sewage samples using a commercial kit (total RNA purification kit by Jena Bioscience® GmbH, Germany). Extracted RNA was first denatured at 90°C for 5 mins before being transcribed into cDNA using random hexamers with SCRIPT cDNA synthesis kit by Jena Bioscience® GmbH, Germany. Amplification of the target DNA sequences for rotavirus VP7 genotypes was done by seminested PCR (snPCR) with first round primers VP7 F 5′- ATGTATGGTATTGAATATACCAC-3′ nucleotide positions 51–71 and VP7 R 5′-AACTTGCCACCATTTTTTCC-3′ nucleotide positions 914–932, [22], inner primers aBT1 5′-CAAGTACTCAATGAATGATGG-3′ nucleotide positions 314–335, ACT2 5′-CAATGATATTAACACATTTTCTGTG-3′ nucleotide positions 411–435, G3 5′-ACGAACTCAACACGAGAGG-3′ nucleotide position 250–269, and aAT8 5′-GTCACACCATTTGTAAATTCG-3′ nucleotide positions 178–198 [23], G9 5′-CTTGATGTGACTAYAAATAC-3′ nucleotide position 757–776, G10 5′-ATGTCAGACTACARATACTGG-3′ nucleotide position 666-687 [24], and G12 5′-GGTTATGTAATCCGATGGCG-3′ nucleotide positions 515–534 [25] as forward primers for the second round reaction and VP7 R as reverse primer [22]. Cycling conditions for first round reaction was 94°C for 2mins, 30 cycles of 94°C for 1min, 42°C for 2mins and 72°C for 1min, and 72°C for 10mins, and second round reaction was the same except number of cycles was reduced to 20 cycles. All PCR products were subjected to electrophoresis using 2% agarose gel and visualized in a gel imaging system (Gel-Doc XR® BioRad, Hercules, California, USA). A gel band size of 619 bp was indicative of a G1 positive sample, 521 bp for G2, 683 bp for G3, 754 bp for G8, 179 bp for G9, 266 bp for G10, and 396 bp for G 12 positive samples.

Amplification of VP4 genotypes was done by seminested PCR of the cDNA using first round primers VP4F 5′-TATGCTCCAGTNAATTGG-3′ nucleotide positions 132–149 and VP4R 5′-ATTGCATTTCTTTCCATAATG-3′ nucleotide positions 775–795 [26], inner primers 2T-1 5′-CTATTGTTAGAGGTTAGAGTC-3′ nucleotide positions 474–494, 1T-1 5′-TCTACTTGGATAACGTGC-3′ nucleotide positions 339–356, and 3T-1 5′-TGTTGATTAGTTGGATTCAA-3′ nucleotide positions 259–278 as reverse primers [27]. VP4F was the forward primer in the second round reaction. Cycling conditions for both reactions was 94°C for 4 mins, 40 cycles of 94°C for 1 min, 45°C for 2 mins and 72°C for 1 min, and final extension of 72°C for 7 mins. A gel band size of 363bp is positive for genotype P[4], 225bp was positive for P[8], and 147bp was positive for genotype P[6].

The NSP4 gene was amplified using primers NSP4F 5-TAAAAGTTCTGTTCCGAGAGAG-3′ forward primer and NSP4 722R 5-TTAAGACCGTTCCTTCCATTAAC-3′ reverse primer [28]. Cycling condition was 95°C for 5 mins, 40 cycles of 94°C for 1 min, 45°C for 2 mins 70°C for 1 min, and 70°C for 7 mins. A gel band size of 752bp was indicative of NSP4 positivity.

### 2.3. Sequencing and Phylogenetic Analysis

Sequencing was done using big dye chemistry on an ABI Prism Genetic Analyzer (Applied Biosystems, Foster, California USA) at Inqaba Bioscience Inc., South Africa, using primers for 2nd round snPCR reactions for VP7 and VP4 amplicons and NSP4 PCR primers.

Sequences were inspected using Chromas Lite Vs 2 (http://www.technelysium.com.au/wp/), and assembled into contigs using Bioedit (www.mbio.ncsu.edu/bioedit/). Strains were determined by BLAST (Basic Local Alignment Search Tool) accessible at http://blast.ncbi.nlm.nih.gov/Blast.cgi from National Center for Biotechnology Information (NCBI) and genotyped using Rota C 2.0. [29]. Sequences were aligned along with other reference rotavirus sequences retrieved from GenBank, using CLUSTAL W program in MEGA 6 software (http://www.megasoftware.net). A Neighbor joining tree was constructed with 1000 bootstrap replicates using Mega 6 software (http://www.megasoftware.net). The sequences of the isolates from this study have been deposited in GenBank with accession numbers KU 866451 to KU 866454 (VP7), KY 964451 to KY 964454 (NSP4), and KY 964455, KY 964456 (VP4).

## 3. Results

**Figure 1:**
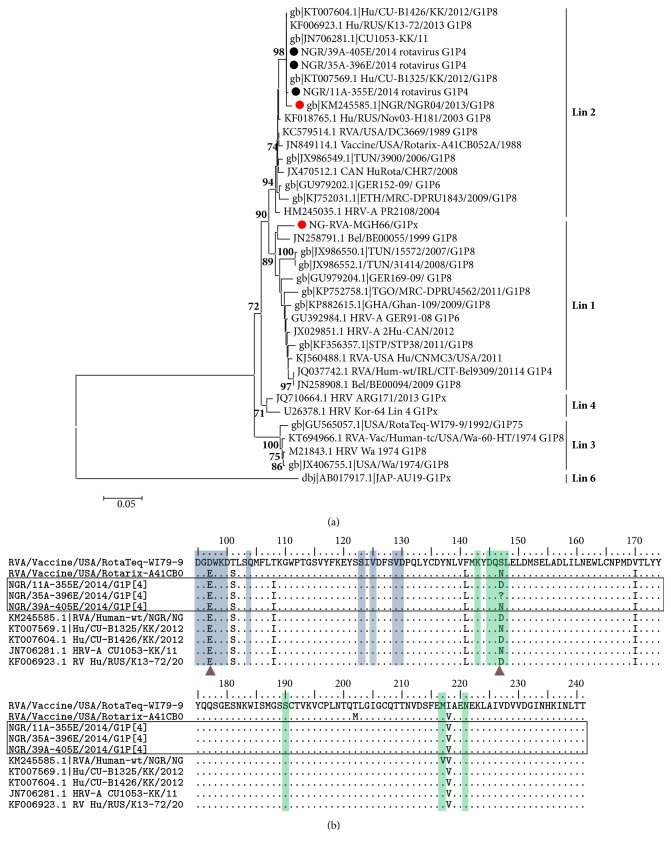
(a) Phylogenetic tree of partial VP7 (575bp) gene sequences of group A rotaviruses. Newly sequenced study strains are shown in black circles; clinical strains from Nigeria are shown by red circles. The GenBank accession numbers are indicated first in the sequence labels, bootstrap values are indicated if ≥ 70%, phylogenetic tree was constructed using the neighbor joining algorithm in MEGA 6.0 with 1,000 bootstrap replicates. Scale bar indicates number of substitutions per site. (b) Alignment of amino acid residues of rotavirus G1 sequences from sewage along Nigeria group A clinical strain NGR04 and Asian strain Hu/Cu-B1 325/KK with vaccine strain (USA/RotaTeq-W179-9/199) as reference. The blue shaded boxes represent 7-1a amino acid residues; green shaded boxes represent 7-2 amino acid residues. The brown triangle represents amino acid substitution within 7-1a and 7-2 antigenic domains.

Seven samples tested positive for rotavirus out of 68 tested (10.3%), genotype G1P[4] had the highest number with five isolates, and genotypes G1P[8] and G3P[6] recorded only one isolate each. After amplicon sequencing, these four gave readable sequences for VP7 (G) gene after purification and sequencing. While only two VP4 (P) gene sequences were readable, four complete NSP4 gene sequences were readable. In this study we analyzed three rotavirus A partial G1 sequences and one G3 sequence identified by seminested RT-PCR from sewage samples obtained in Nigeria in 2014. Sequence data were analyzed using BLAST search and Rota C software. Three G1 sequences showed high amino acid sequence identity of 99.1 to 100% in their partial VP7 codding sequence to three Asian strains isolated from China, CU.1053/G1P[8], CU.B1426/G1P[8], and CU.B1325/G1P[8] and one isolate from Russia RUS/K13-72 (Table 1). The three environmental strains analyzed also showed very high amino acid similarity with 98.1 to 99.1% sequence similarity to a recently isolated rotavirus strain from Maiduguri Northern Nigeria NGR-04/ G1P[8] (Table 1). Phylogenetic relationship to other reference strains also showed that the three environmental strains isolated in this study clustered closely together within Lineage 2 along with Chinese isolates GenBank accession numbers JN70628.1, KT007604.1, and KT007569.1 and Russian isolate GenBank accession number KF006923.1, with high bootstrap value 99% (Figure 1(a)). Our sequences also clustered closely along with a Nigerian G1 isolate NGR-04/ G1P[8] isolated in 2013 GenBank accession KM245585.1, and also with a Russian isolate RUS/Nov03-H181 GenBank accession number KF018765.1. Amino acid alignment of study G1 sequences with the full coding sequence of Rotarix G1P[8] vaccine strain showed conserved amino acid (aa) residues within the 7-1a (aa positions 95-100, 104, 123, 125, and 129-130) and 7-2 (aa positions 143-148, 190, 217, and 221) antigenic domains among our environmental isolates. Amino acid substitutions were however observed among our study strains in reference to vaccine strain RotaTeq-W179-9 within the 7-1a antigenic domain region, at amino acid position 97 (D-97→E) and at antigenic domain region 7-2 with substitutions S-147→D and S-147→N (Figure 1(b)).

**Figure 2:**
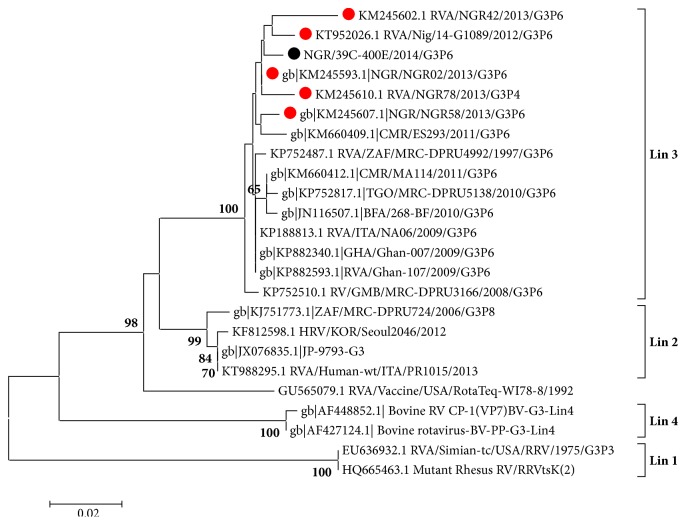
Phylogenetic tree of partial VP7 (625bp) gene sequence of group A rotaviruses. Newly sequenced study strain is shown in black circle; clinical strains from Nigeria are shown in red circles. The GenBank accession numbers are indicated first in the sequence labels, bootstrap values are indicated if ≥ 70%, and phylogenetic tree was constructed using the Neighbor joining algorithm in MEGA 6.0 with 1,000 bootstrap replicates.

Figure 2 shows the phylogenetic relatedness study G3 sequence with other representative G3 strains from GenBank. The Nigerian G3 sequence (KU866454) coclustered together with other West African isolates with a high bootstrap value of 100%, within Lineage 3 of genotype G3.

**Figure 3:**
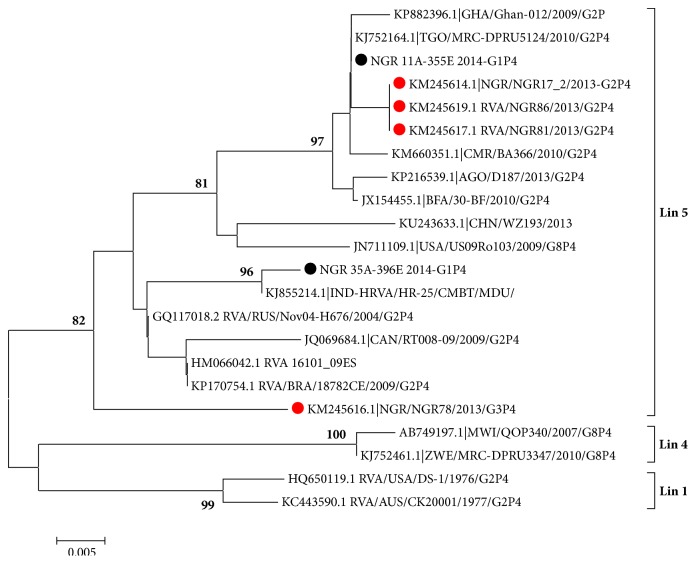
Phylogenetic tree of partial VP4 (320bp) gene sequence of group A rotaviruses. Newly sequenced study strains are shown in black circles; clinical strains from Nigeria are shown in red circles. The GenBank accession numbers are indicated first in the sequence labels, and bootstrap values are indicated if ≥ 60%.

**Figure 4:**
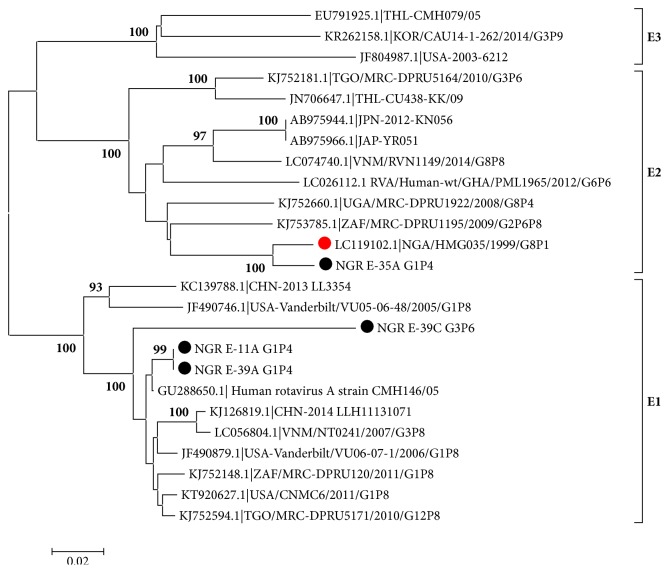
Phylogenetic tree of complete NSP4 (720bp) gene sequence of group A rotaviruses. Newly sequenced study strains are shown in black circles; clinical strains from Nigeria are shown in red circles. The GenBank accession numbers are indicated first in the sequence labels, and bootstrap values are indicated if ≥ 60%. Scale bar indicates number of substitutions per site.

Phylogenetic analysis of the two study P[4] sequences belonging to isolates NGR E-11A and NGR E-35A (Figure 3) shows that they cluster into genotype P[4] Lineage 5 along with several West African isolates including clinical isolates recovered from children with AGE during a 2013 rotavirus outbreak in Nigeria [13]. One of the P[4] isolates clustered very closely with an isolate from India (GenBank accession no.: KJ855214.1). Phylogenetic analysis of study NSP4 sequences (Figure 4) revealed that isolate 35A belonged to genotype E2, while others (11A, 39A, and 39C) belonged to E1.  Table 2 shows the VP7, VP4, and NSP4 genotype assignments of rotavirus group A isolates from sewage in Nigeria 2014.

## 4. Discussion

The current study characterized three G1, sequences NGR/11A, NGR/35A, and NGR/39A, and one G3 sequence, NGR/39C, recovered from sewage in Northern Nigeria. Previous studies have reported both genotypes in stool specimens of children with gastroenteritis from different parts of Nigeria [11, 12, 14]. Genotypes G1P[4] and G1P[8] have consistently been identified as the predominant circulating genotype combination responsible for rotavirus outbreaks in Nigeria [11, 12]. However recent studies have identified the emergence of genotype G12 among children suffering from rotavirus induced gastroenteritis in South West Nigeria [15]. Phylogenetic analysis of the 620bp partial VP7 genes from our study revealed that they all fall within Lineage 2 of genotype G1 (Figure 1(a)), clustering together with three Asian rotavirus strains from China {Cu-1053-KK, Hu/Cu-B1426/KK, and Cu-B1325/KK} and one Russian strain {RUS/K12-72}, and our isolates also clustered with a recent Nigerian isolate recovered from a diarrheic child GenBank accession number KM 245585.1. Amino acid similarity values of our study sequences with the three Chinese isolates gave between 99.1% and 100% amino acid sequence similarities, serving as evidence of the possibility of a common ancestral origin of these viruses. The identification of our G1 rotavirus genotypes in sewage also supports the fact that this virus could have been responsible for past outbreaks and is currently circulating among the general population in Nigeria. This is further supported by the fact that a Nigerian clinical isolate recovered in 2013 from a diarrheic child [30], also clustered closely with our environmental isolates (Figure 1), buttressing the fact that they could be of common parental origin with the Chinese strains. Prior to this report, the only Lineage 2 rotavirus G1 strain was detected in a child in Northern Nigeria in 2013 [30]. A limitation to this study however is the small number of sequences analyzed due to the few number of RVA detections and low amplicon yield of some samples. Analysis of the amino acid residues of the surface exposed of our study strains was homologous with reference vaccine strain Rotarix-A41CB0. Amino acid substitutions were however observed among our study strains in reference to vaccine strain RotaTeq-WI79-9 within the 7-1a antigenic domain region, at amino acid position 97 (D-97→E) and at antigenic domain region 7-2 with substitutions S-147→D and S-147→N (Figure 1(b)). The mutations on these sites are not synonymous with immune escape and show that wild type G1 strains circulating in Nigeria are likely to be susceptible to neutralization by antibodies to existing vaccine strains. Analysis of a 680 partial VP7 sequence was identified as genotype G3, and phylogenetic analysis of our G3 isolate (accession no: KU866454) showed that it coclustered within Lineage 3, together with other Nigerian clinical rotavirus isolates recovered during the 2013 outbreak [30], as well as other strains from West Africa (Figure 2). Interestingly the West African clinical isolates including our environmental isolate formed a cluster with 100% bootstrap value (Figure 2). The identification of this unique cluster serves as evidence of circulation of genotype G3 Lineage 3 throughout West Africa.

Further characterization of our sewage isolates by VP4 gene sequencing and phylogenetic analysis showed that the two isolates analyzed coclustered within Lineage 5 along with other isolates from Nigeria, Brazil, Russia, and some West African countries. One of our G1P[4] isolates NGR E-35A clustered very closely with an Indian isolate (KJ855214.1) with 95% bootstrap value as shown in Figure 3. Isolate NGR E-11A clustered with largely Nigerian clinical isolates recovered during the 2013 outbreak [30]. This observation shows the clinical relevance of environmental surveillance, going by the fact that the study isolate has the potential to cause future outbreaks, if sewage effluent containing this virus contaminates food or drinking water sources. Figure 4 shows the results of phylogenetic analysis of the NSP4 gene sequences of study isolates and reveals that one isolate 35A clustered within genotype E2, while the three other isolates 11A, 39A, and 39C clustered within genotype E1 (Table 2). The genotypes detected in this study belong to mostly modern lineages as previously reported [30]. This is expected because viruses recovered from this study are composed of mostly recently shed viruses by both symptomatic and asymptomatic rotavirus infected individuals. However what is of concern is that almost all the genotypes detected have been linked to genotypes that have caused outbreaks in Nigeria [12, 30, 31]. This shows the need of continuous molecular surveillance of this kind to serve as a cheaper and easier option of rotavirus strain characterization and monitoring.

## 5. Conclusion

Our study has characterized VP7, VP4, and NSP4 gene sequences of rotavirus from sewage demonstrating the phylogenetic relationship to other representative rotavirus strains from neighboring West Africa and representative isolates from outside the African continent. We have also reported the intragenotype diversity of identified G1 and G3 sequences as well as P[4] and NSP4 genes from environmental isolates in Nigeria. We established the circulation of G1P[4] strains with close VP7 gene sequence similarity with group A rotavirus G1 from Asia. We have also identified the possibility of regionally confined G3 Lineage 3 strains circulating within West Africa. These findings highlight the importance of molecular epidemiology of rotavirus particularly from environmental sources such as sewage in the identification of emerging rotavirus strains to augment rotavirus disease prevention and control.

## Figures and Tables

**Table 1:** Comparison of amino acid sequence similarity between Nigerian environmental rotavirus A,VP7 (G1P[4]) gene sequences from sewage and other reference RVA G1 sequences from Asia and West Africa, analyzed in this study including representative G1P[8] vaccine strain.

**Reference strains**	**Environmental RVA G1P**[4]** isolates from this study**		
	11A	35A	39A
**CU.1053/ G1P**[8]** (Asian)**	99.1%	100%	100%
**CU.B1426/ G1P**[8]** (Chinese)**	99.1%	100%	100%
**CU.B1325/ G1P**[8]** (Chinese)**	99.1%	100%	100%
**NGR-04/ G1P**[8]** (Nigerian)**	98.1%	99.1%	99.1%
**MGH66/ G1Px (Nigerian)**	87.7%	88.7%	88.7%
**TGO/ DPRU4562 G1P**[8]	86.7%	87.7%	87.7%
**STP38/G1P**[8]** (W.Afr)**	87.7%	88.7%	88.7%
**Gha-109/ G1P**[8]**(W.Afr)**	87.7%	88.7%	88.7%
**RotaTeq/WI79.9/G1P**[75]**(Vac)**	84.9%	85.8%	85.8%
**USA/Wa/ G1Px (Vac)**	85.8%	86.8%	86.8%

N.B: W.Afr represents West Africa. Vac represents vaccine strain.

**Table 2:** Genotype assignments of VP4, VP7, and NSP4 gene sequences of group A rotavirus isolated from sewage in Nigeria 2014.

**Isolate name**	**Genotypes**		
	**VP7(Lin)**	**VP4 (Lin)**	**NSP4**
**E-11A**	G1 (Lin 2)	P[4] (Lin 5)	E1
**E-35A**	G1 (Lin 2)	P[4] (Lin 5)	E2
**E-39A**	G1 (Lin 2)	P[4] (NA)	E1
**E-39C**	G3 (Lin 3)	P[6] (NA)	E1

NB: Lin represents lineage. NA represents not available.

## Data Availability

The data used to support the findings of this study are available from the corresponding author upon request.
